# The Role of ROS Homeostasis in ABA-Induced Guard Cell Signaling

**DOI:** 10.3389/fpls.2020.00968

**Published:** 2020-06-30

**Authors:** Anthony E. Postiglione, Gloria K. Muday

**Affiliations:** Department of Biology and the Center for Molecular Signaling, Wake Forest University, Winston Salem, NC, United States

**Keywords:** guard cell, reactive oxygen species, stomata, abscisic acid, flavonols, respiratory burst oxidase homolog

## Abstract

The hormonal and environmental regulation of stomatal aperture is mediated by a complex signaling pathway found within the guard cells that surround stomata. Abscisic acid (ABA) induces stomatal closure in response to drought stress by binding to its guard cell localized receptor, initiating a signaling cascade that includes synthesis of reactive oxygen species (ROS). Genetic evidence in Arabidopsis indicates that ROS produced by plasma membrane respiratory burst oxidase homolog (RBOH) enzymes RBOHD and RBOHF modulate guard cell signaling and stomatal closure. However, ABA-induced ROS accumulates in many locations such as the cytoplasm, chloroplasts, nucleus, and endomembranes, some of which do not coincide with plasma membrane localized RBOHs. ABA-induced guard cell ROS accumulation has distinct spatial and temporal patterns that drive stomatal closure. Productive ROS signaling requires both rapid increases in ROS, as well as the ability of cells to prevent ROS from reaching damaging levels through synthesis of antioxidants, including flavonols. The relationship between locations of ROS accumulation and ABA signaling and the role of enzymatic and small molecule ROS scavengers in maintaining ROS homeostasis in guard cells are summarized in this review. Understanding the mechanisms of ROS production and homeostasis and the role of ROS in guard cell signaling can provide a better understanding of plant response to stress and could provide an avenue for the development of crop plants with increased stress tolerance.

## Introduction

Stomatal aperture must be tightly regulated to ensure optimal CO_2_ entry for photosynthesis while protecting plants against excess water loss and pathogen attack (Nilson and Assmann, 2007). The opening and closing of stomata are mediated by changes in turgor pressure inside guard cells that surround the stomatal pore (Schroeder et al., 2001). Guard cell turgor is controlled by signal transduction cascades that are induced by many environmental signals, including water availability (Xu et al., 2014; Xu et al., 2016; Li et al., 2017; Qi et al., 2018; Qu et al., 2018; Tõldsepp et al., 2018). Decreased water availability increases abscisic acid (ABA) synthesis, which induces stomatal closure and a myriad of other plant responses (Zhu, 2016; Vishwakarma et al., 2017).

**Figure 1:**
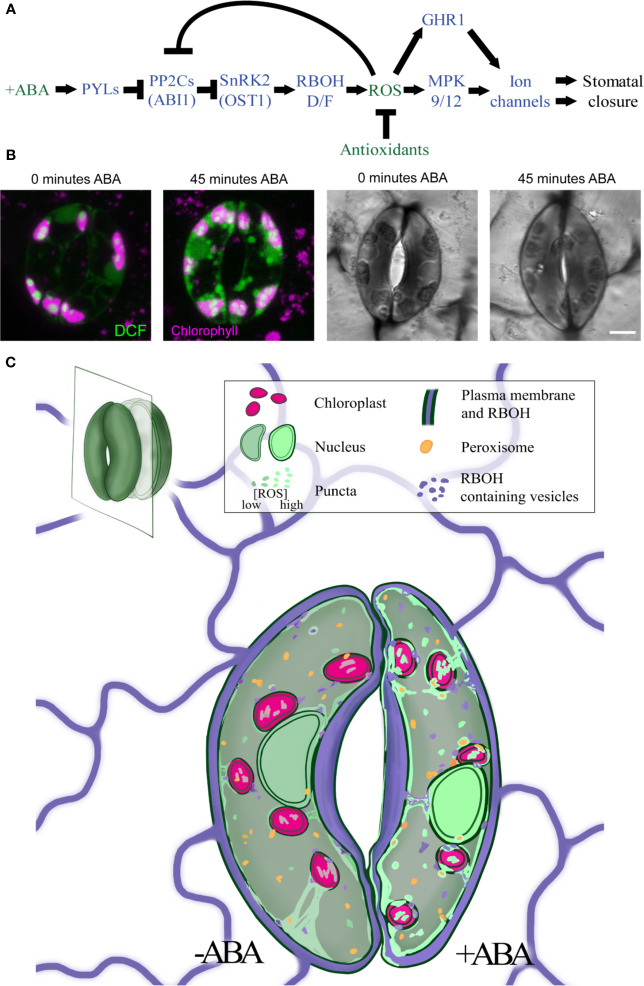
ABA increases ROS levels in guard cells in multiple subcellular locations. **(A)** A schematic model of the ABA signaling pathway during stomatal closure. Blue fonts represent proteins, while green fonts represent molecules. **(B)** Treatment with ABA for 45 min increases DCF fluorescence and decreases stomatal aperture in tomato guard cells. **(C)** The signal of the generic ROS sensor, DCF (green), is detected in the cytosol, nucleus, chloroplasts (pink), and endomembranes, with rapid and dramatic increases in all these locations in response to ABA treatment. RBOH enzymes (purple) produce ROS at the plasma membrane but can be internalized into endosomes. ROS signal also overlays peroxisome (orange), but whether this signal increases with ABA has not yet been reported. Central vacuole is not shown in illustration.

Guard cells have an elegant signaling cascade induced upon ABA binding to a family of soluble receptor proteins including PYRABACTIN RESISTANCE 1 (PYR1) (Park et al., 2009), which is summarized in **Figure 1A**. The ABA-bound receptor inhibits Clade A protein phosphatases type 2C (PP2Cs), such as ABI1 (Ma et al., 2009; Park et al., 2009; Nishimura et al., 2010). The inhibition of PP2Cs prevents protein dephosphorylation and negative regulation of ABA signaling (Park et al., 2009). Targets of PP2Cs include Sucrose nonfermenting Related Kinase 2 family members (SnRK2s), with the best characterized target being OPEN STOMATA 1 (OST1)/SnRK2.6 (Mustilli et al., 2002). OST1 transmits the ABA signal through phosphorylation of downstream targets, ultimately triggering a rapid burst of Reactive Oxygen Species (ROS) (Pei et al., 2000), which can then stimulate guard cell ion channels (Geiger et al., 2009; Demidchik, 2018). In this review, we summarize the current literature on ABA-induced ROS production, targets of ROS signaling pathways, and how ROS homeostasis is maintained to keep ROS concentration appropriate for productive guard cell signaling.

## ABA Induced ROS in Guard Cells Drives Stomatal Closure

ROS can act as a developmental and hormonal response signal (Mittler, 2017; Huang et al., 2019) with ABA-induced ROS bursts emerging as an elegant example (Singh et al., 2017). ABA-induced ROS accumulation in guard cells is most frequently visualized by fluorescence of the ROS sensor 2′,7′-dichlorofluorescein (DCF) (Pei et al., 2000; Murata et al., 2001; Watkins et al., 2014; Watkins et al., 2017). Improvements in the resolution of confocal microscopy have revealed ABA-induced ROS signals in the guard cell nucleus, cytoplasm, chloroplasts, and endomembrane bodies (Watkins et al., 2017), (**Figure 1B**). ABA-dependent increases in DCF fluorescence are rapid, having been reported within 2 min after ABA treatment (Pei et al., 2000), although other studies detect slower changes observed within 15 min and maximal after 45 min (**Figure 1B**) (Watkins et al., 2017). These DCF changes are slower than changes in ion movements detected at 1 min after ABA treatment *via* electrophysiology (Hamilton et al., 2000). This difference may reflect the methodology used for these measurements.

**Figure 2:**
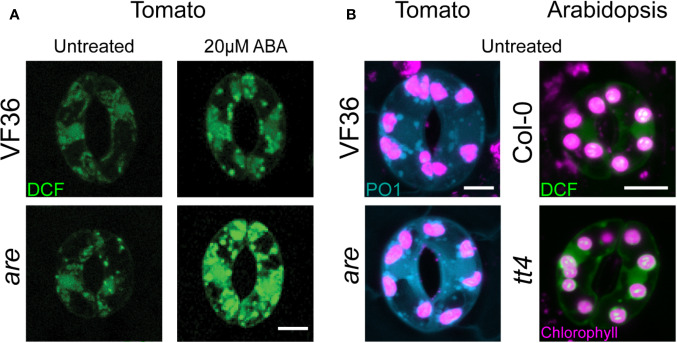
Arabidopsis and tomato mutants with decreased flavonoid antioxidants have increased ROS accumulation. **(A)** Confocal micrographs of DCF-stained guard cells of 4-week-old VF36 (wild-type) and *are* plants show that flavonol deficient mutants have increased ROS levels both in the absence or presence of 20 µM ABA. **(B)** Confocal micrographs of DCF and PO1 fluorescence in guard cells of 4-week-old tomato and Arabidopsis leaves show tomato and Arabidopsis with decreased flavonol levels have increased total ROS and H_2_O_2_ levels. Scale bars = 5 µm. DCF signal is shown in green, PO1 signal in blue, and chlorophyll autofluorescence in magenta. Images obtained from experiments completed in (Watkins et al., 2014; Watkins et al., 2017).

It is important to understand which ROS are increased in response to ABA as they each have distinct functions. ROS as they each have distinct functions are highly reactive derivatives of molecular oxygen, which include hydroxyl radical (**·**OH), singlet oxygen (^1^O_2_), superoxide anion (O_2_**·^−^)**, and hydrogen peroxide (H_2_O_2_). DCF is a general ROS sensor as it is oxidized by multiple ROS (Chen et al., 2010). A recent study used the H_2_O_2_ specific probe Peroxy Orange1 (PO1) and showed that the pattern of H_2_O_2_ accumulation parallels the total ROS profile detected by DCF, except in the nucleus where little PO1 signal was observed (Watkins et al., 2017) (**Figure 2B**).

ABA induced stomatal closure has been shown to be dependent on ROS increases using several approaches. Guard cell closure is reduced, but not totally abolished, by treatment with ROS scavengers (Zhang et al., 2001) and inhibitors of or mutants in ROS producing enzymes (Pei et al., 2000; Watkins et al., 2017; Iwai et al., 2019). These partial effects are consistent with ROS independent closure and/or multiple sources of guard cell ROS. The most intriguing results were obtained *via* genetic mutants of Respiratory Burst Oxidase Homolog (RBOH)/NADPH Oxidase (NOX) enzymes, suggesting RBOH dependent ROS synthesis drives the ABA response (Kwak et al., 2003).

## Expression and Function of RBOH Enzymes in ABA Signaling

The *Arabidopsis* genome encodes 10 RBOH family members, RBOHA-RBOHJ that have important roles in signaling induced ROS synthesis (Suzuki et al., 2011; Chapman et al., 2019). RBOH enzymes have distinct expression patterns and regulate development and signaling (Chapman et al., 2019). These plasma membrane (PM)-localized proteins have six transmembrane domains, a C-terminal FAD-binding domain and two N-terminal calcium-binding EF hands (Torres and Dangl, 2005). RBOHs produce extracellular superoxide by transferring electrons from NADPH or FADH_2_ to oxygen (Suzuki et al., 2011). Superoxide can then be converted to H_2_O_2_ spontaneously or by Superoxide Dismutase (SOD). This extracellular H_2_O_2_ enters the plant cells through PM aquaporins (Bienert et al., 2007; Tian et al., 2016; Rodrigues et al., 2017).

Genetic approaches have demonstrated the importance of RBOHD and RBOHF in ABA-induced stomatal closure (Kwak et al., 2003). An *rbohf* single mutant and an *rbohd/f* double mutant show reduced rates of ABA-induced ROS synthesis and partial impairment in stomatal closure, while the *rbohd* single mutant had wild-type responses (Kwak et al., 2003). Treatment with an RBOH inhibitor, diphenyleneiodonium (DPI), also impaired ROS production and ABA-induced stomatal closure in Arabidopsis (Zhang et al., 2001) and tomato (Watkins et al., 2017). Together these results implicate RBOHs as important modulators of ABA-dependent stomatal closure.

## Regulation of RBOH Synthesis and Activity

The synthesis of RBOHs is regulated transcriptionally (Yun et al., 2011; Hao et al., 2014; Qu et al., 2017). In Arabidopsis, *RBOHD* and *RBOHF*, the two RBOHs with known function in guard cells, are expressed in this cell type as judged by transcript abundance and transcriptional reporters (Kwak et al., 2003; Chapman et al., 2019). The abundance of *RBOHD* and *RBOHF* transcripts has been reported to be increased by ABA treatment and abiotic stress including drought, salt, and elevated osmoticum (Kwak et al., 2003; Kilian et al., 2007).

RBOH activity is also posttranslationally regulated to coordinate the timing and magnitude of the ROS burst. Mutants in *phospholipase Dα1* (*PLDα1*) have decreased phosphatidic acid synthesis, which impaired ABA-induced ROS production and stomatal closure (Zhang et al., 2009). Treatment with DPI did not affect the *plda1* mutant further, implicating phosphatidic acid as a positive regulator of RBOHs (Zhang et al., 2009). RBOHF is regulated by Ca^2+^ dependent phosphorylation by Calcineurin B-Like (CBL) which interacts with CBL-Interacting Protein Kinases (CIPKs) (Kimura et al., 2013). One report indicated that this complex negatively regulated ROS synthesis (Kimura et al., 2013), while a second report showed that coexpression of *CIPK26* and *CBL1* or *CBL9* increased RBOHF-dependent ROS production, while *CIPK26* alone had no effect (Drerup et al., 2013). Additionally, activated OST1 phosphorylates RBOHF, which may be required for its activation (Sirichandra et al., 2009). Nitrosylation of RBOHD at Cysteine 890 eliminates ROS production to block guard cell apoptosis when these cells are undergoing a pathogen induced immune response in Arabidopsis (Yun et al., 2011).

## ROS Integration with ABA Signaling Machinery

A central question is where ABA-induced ROS integrates with guard cell signaling machinery. Two PP2C enzymes, ABA insensitive 1 (ABI1) and ABI2, which were identified in mutant screens for ABA insensitivity in stomatal closure assays, are negative regulators of ABA signaling. Both proteins are inactivated by H_2_O_2_ (Meinhard and Grill, 2001; Meinhard et al., 2002). The *abi1-1* and *abi2-1* mutants are defective in interactions with ABA receptors resulting in constitutive SnRK2 inactivation, blocking ABA signaling and stomatal closure (Umezawa et al., 2009). In *abi1-1*, ABA treatment failed to induce ROS production and stomatal closure (Murata et al., 2001). Impaired stomatal closure in *abi1-1* was restored by exogenous H_2_O_2_ treatment, suggesting that ROS is downstream of ABI1 (**Figure 1A**). However, in this same study, ABA induced a ROS burst in *abi2-1*, and the impaired stomatal closure was not rescued by H_2_O_2_ treatment, suggesting ABI2 may be downstream of the ABA-induced ROS burst.

ROS can regulate plant signaling cascades by modulating the activity of target proteins through reversible oxidation of cysteine residues (Waszczak et al., 2015). ABA-induced ROS activates the plasma membrane-localized Guard Cell Hydrogen Peroxide Resistant1 (GHR1) receptor-like kinase which controls calcium channel activation (Pei et al., 2000; Hua et al., 2012; Sierla et al., 2018). Mitogen Activated Protein Kinases (MAPKs) are also downstream targets of ROS in guard cells (Lee et al., 2016). Treatment with both ABA and H_2_O_2_ activated the guard cell specific MPK12, which works with MPK9 to positively regulate ABA-induced stomatal closure (Jammes et al., 2009). Two other guard cell map kinases, MPK3 and MPK6, are implicated in guard cell response to pathogen attack and have increased activity after H_2_O_2_ treatment (Kovtun et al., 2000; Yuasa et al., 2001). MPK3 and MPK6 activation is unaffected in the *rbohd/f* double mutant following treatment with flg22, a pathogen elicitor, suggesting that MPK3/MPK6 activation during pathogen response is not RBOH dependent (Xu et al., 2014). Altogether, the current evidence suggests that ABI1 is upstream of ABA-induced ROS synthesis (and feedback inhibited by ROS) while ABI2, GHR1, and MAPKs are downstream of ROS (**Figure 1A**), although aspects of this model need further experimentation.

## Antioxidants Regulate ROS Homeostasis to Modulate Stomatal Aperture

For ROS to serve as productive signaling molecules, they need to increase to drive signaling and development (Chapman et al., 2019), but if ROS increases are left unchecked, ROS can accumulate to dangerous levels resulting in oxidative damage of proteins, DNA, and lipids (Betteridge, 2000). Guard cells contain both enzymatic and nonenzymatic machinery to maintain ROS homeostasis to prevent ROS from reaching damaging levels (Chen and Gallie, 2004; Watkins et al., 2014; Singh et al., 2017; Watkins et al., 2017; Yamauchi et al., 2019). These antioxidants regulate the responses to ABA in guard cells.

Flavonols are a class of plant specialized antioxidant metabolites that reduce ROS accumulation in guard cells to regulate stomatal closure (Watkins et al., 2014; Watkins et al., 2017). Mutants in tomato and Arabidopsis with decreased flavonol production had increased guard cell ROS (Watkins et al., 2014) and were more sensitive to ABA-induced stomatal closure (Watkins et al., 2017) (**Figure 2**). Mutants with elevated levels of flavonols have decreased ROS accumulation and decreased ABA sensitivity (Watkins et al., 2017) as visualized using DCF and PO1. ROS signals in flavonol deficient mutants were increased in chloroplasts and unidentified endomembrane structures with both sensors, while DCF, but not PO1, was elevated in the nucleus (**Figure 2**).

Guard cells also maintain ROS homeostasis during ABA signaling *via* antioxidant enzymes such as catalases, SODs, thioredoxin reductases, glutathione peroxidases, and ascorbate peroxidases (APX) (Chen and Gallie, 2004; Miao et al., 2006; Jannat et al., 2011; Tiew et al., 2015). The H_2_O_2_ scavengers, APX1 and catalases 1 and 3 are abundant enzymatic antioxidants in guard cells (Chen and Gallie, 2004; Jannat et al., 2011) and mutants deficient in these enzymes have enhanced ABA responses (Pnueli et al., 2003; Jannat et al., 2011). The *calmodulin-like20* (*cml20*) mutant showed decreased APX2 expression and increased ROS levels, resulting in an ABA hypersensitive stomatal phenotype (Wu et al., 2017), consistent with the absence of antioxidant enzyme synthesis.

## Distinct Locations of ABA-Induced ROS

ABA-induced ROS accumulates in the cytoplasm, chloroplasts, nucleus, and endomembrane structures; many of these locations are highlighted by DCF and PO1 accumulation shown in **Figures 1** and **2** (Watkins et al., 2017). In addition to membrane-localized RBOHs, chloroplasts and peroxisomes are also major sources of plant cell ROS that are produced by organelle-localized metabolic processes and enzymatic machinery (Foyer and Noctor, 2003; Asada, 2006). The following sections explore organelle specific ABA-regulated ROS accumulation and signaling.

### Nuclear ROS

ABA treatment increases ROS levels in guard cell nuclei (Leshem et al., 2010; Watkins et al., 2014; Watkins et al., 2017), although the mechanisms for this increase are unknown. Isolated tobacco nuclei have increased H_2_O_2_ following calcium application (Ashtamker et al., 2007), consistent with ROS synthesized within this organelle. Nuclear ROS may also increase *via* diffusion from the cytosol, retrograde signal transport from other organelles (see *Chloroplast* section), or through trafficking of ROS-producing enzymes to the nucleus. The mammalian NOX1 and NOX4 localize to the nucleus to produce ROS necessary for regulating gene expression, (Chamulitrat et al., 2003; Kuroda et al., 2005; Saez et al., 2016), but whether plants share this mechanism is unclear.

Nuclear ROS may function to regulate transcriptional cascades through reversible cysteine oxidation of transcription factors (TFs) to change their activity and/or localization (Peleg-Grossman et al., 2010; Poole, 2015). In plants, several stress responsive TF families such as WRKY, MYB, NAC, heat shock factors (HSF), and ZAT are redox regulated (He et al., 2018). Additionally, ABA-induced ROS could function to oxidize proteases that degrade TFs or modulate kinase or phosphatase activity that target TFs through mechanisms that have been demonstrated in other systems (Schieber and Chandel, 2014).

### Endosomes and Endomembrane Trafficking

ABA increases ROS in small endomembrane structures, visualized with both DCF and PO1, which share common features with endosomes (Leshem et al., 2010; Hao et al., 2014; Watkins et al., 2017). In mammalian systems, redox-activated endosomes, termed “redoxosomes”, contain NOX family components that deliver ROS where it is needed for productive signaling (Oakley et al., 2009). While literature surrounding endosomal ROS in plants is scarce, endomembrane trafficking has been shown to play a role in ABA-induced stomatal closure. Knockdown of vesicle associated membrane protein 71 family (VAMP71), which mediates endosome fusion to the central vacuole, resulted in increased quantities of ROS-containing vesicles in the cytoplasm, although the magnitude of total ROS was similar to the wild-type. VAMP71 knockdown also impaired stomatal closure following ABA treatment (Leshem et al., 2010). RBOH trafficking may drive these localized ROS increases.

ABA treatment results in clathrin-dependent endocytosis of GFP-RBOHD (Hao et al., 2014). Similarly, salt stress resulted in RBOH endocytosis and increased ROS levels in endosomes (Leshem et al., 2007). Trafficking of ion channels to and from the PM regulates guard cell turgor to modulate stomatal opening (Meckel et al., 2004). Together these findings suggest that the internalization of RBOHs and ion channels into endosomes may be a mechanism to spatially regulate intracellular ROS-mediated signaling. In tomato guard cells, ABA was shown to induce unidentified ROS-containing endomembrane structures that were in greater quantities in mutants with reduced synthesis of flavonol antioxidants (Watkins et al., 2017). Determination of the organelle identity of these endomembrane structures is an important area of future study.

Two other endomembrane organelles may participate in ROS signaling in guard cells. The guard cell central vacuole has recently been suggested to be a site of ROS synthesis *via* a copper amine oxidase (CuAO*δ*) involved in ABA-dependent vacuolar ROS increases and stomatal closure (Fraudentali et al., 2019). It is not yet clear how vacuolar ROS signals integrate into ABA signaling and guard cell closure. Guard cell ROS may also be regulated by autophagy of aggregated peroxisomes formed under oxidative stress (Yamauchi et al., 2019). Autophagy impaired mutants had increased ROS levels, increased number of oxidized peroxisomes, and decreased sensitivity to light-dependent stomatal opening. Antioxidant treatments rescued this phenotype (Yamauchi et al., 2019), yet whether this is linked to ABA-induced closure was not reported.

### Chloroplasts

ABA biosynthesis begins in chloroplasts (Finkelstein, 2013) and ABA signaling and ROS production also occur in this organelle (Asada, 2006; Pornsiriwong et al., 2017). Mg-chelatase H subunit (CHLH) is a chloroplast protein that functions in communicating chloroplast signals to the nucleus, or retrograde signaling, and positively regulating ABA signaling. A *CHLH* RNAi line has impaired stomatal closure and drought tolerance (Shen et al., 2006). It is still unclear whether CHLH affects ABA-induced ROS production, though *OST1* expression was decreased following CHLH knockdown, suggesting there is crosstalk with positive regulators of ABA-induced ROS (Shen et al., 2006). Initially CHLH was suggested to act as an ABA receptor, but was later shown to modulate ABA signaling without binding ABA directly (Tsuzuki et al., 2011) suggesting additional studies are needed (Cutler et al., 2010).

Other signals that originate within guard cell chloroplasts have been shown to stimulate ROS increases and stomatal closure in Arabidopsis (Zhao et al., 2019). The molecule 3′-phosphoadenosine 5′-phosphate (PAP) shows oxidative stress-induced synthesis and restores ABA-induced ROS production and stomatal phenotypes to the ABA insensitive mutants *ost1-*2 and *abi1-1*. PAP may function in parallel to the canonical ABA machinery by upregulating Ca^2+^ signaling proteins that activate SLAC1 and other ion channels to regulate stomatal closure (Pornsiriwong et al., 2017).

H_2_O_2_ can be a chloroplast retrograde signal by moving through stromules, which are tubules that extend from chloroplasts to the nucleus (Kwok and Hanson, 2004; Caplan et al., 2015) at a sufficient concentration to induce programmed cell death during pathogen response (Caplan et al., 2015). Stromule formation was induced by ABA and other oxidative signals (Gray et al., 2012; Brunkard et al., 2015), but whether stromules can transport ROS to the nucleus in other stress responses still needs to be evaluated. ROS produced through photorespiration have also been shown to be necessary for stomatal closure (Iwai et al., 2019). Treatment with two photosynthetic electron transport inhibitors in Arabidopsis led to reduced guard cell ROS and stomatal closure in the wild-type and the *rbohd/f* double mutant (Iwai et al., 2019). Together these findings highlight the importance of chloroplast signaling on ABA-induced ROS production and stomatal closure.

## Conclusion and Future Directions

It is an exciting time to study the role of ROS as second messengers in guard cell signaling. Genetic approaches have shown that ROS produced by RBOH enzymes at the PM plays a significant role in ABA-induced stomatal closure, although there may be other ROS sources that regulate this response. Changes in ROS levels in many of the organelles discussed in this review have been identified *via* colocalization of ROS dyes with organelle-specific probes and reporters. ROS accumulation and how it changes in guard cells in response to elevated ABA in nuclei, chloroplasts, and endosomes are illustrated in **Figure 1C**, which synthesizes results from multiple experiments summarized in this review. While fluorescent dyes provide beneficial spatial information for ROS levels, limitations such as dye irreversibility, working concentration, and incubation time for uptake restrict insights into the temporal dynamics of ROS regulation in guard cells. Moving forward, it will be valuable to apply new tools such as organelle localized, genetically encoded ROS sensors to fully elucidate this information (Anjum et al., 2020). Mutants defective in ROS synthesis and signaling and antioxidant synthesis highlight roles for each organelle in ROS-dependent stomatal closure. Defining the contribution of each ROS source to ABA signaling will allow better understanding of how ABA-induced ROS signals are generated, communicated, and balanced in the guard cell signaling circuit.

## Author Contributions

AP wrote and edited the article. GM provided scientific and editorial input.

## Funding

This work was supported by a grant from the National Science Foundation IOS-1558046 to GM and a Center for Molecular Signaling Graduate Research Fellowship to AP.

## Conflict of Interest

The authors declare that the research was conducted in the absence of any commercial or financial relationships that could be construed as a potential conflict of interest.
